# RNY (YRNA)-derived small RNAs regulate cell death and inflammation in monocytes/macrophages

**DOI:** 10.1038/cddis.2016.429

**Published:** 2017-01-05

**Authors:** Zoheir Hizir, Silvia Bottini, Valerie Grandjean, Michele Trabucchi, Emanuela Repetto

**Affiliations:** 1 INSERM U1065, Centre Méditerranéen de Médecine Moléculaire (C3M), Team 10 'Control of Gene Expression', Nice, France; 2 University of Nice Sophia Antipolis, Faculty of Medicine, Nice, France

## Abstract

The recent discovery of new classes of small RNAs has opened unknown territories to explore new regulations of physiopathological events. We have recently demonstrated that RNY (or Y RNA)-derived small RNAs (referred to as s-RNYs) are an independent class of clinical biomarkers to detect coronary artery lesions and are associated with atherosclerosis burden. Here, we have studied the role of s-RNYs in human and mouse monocytes/macrophages and have shown that in lipid-laden monocytes/macrophages s-RNY expression is timely correlated to the activation of both NF-*κ*B and caspase 3-dependent cell death pathways. Loss- or gain-of-function experiments demonstrated that s-RNYs activate caspase 3 and NF-*κ*B signaling pathways ultimately promoting cell death and inflammatory responses. As, in atherosclerosis, Ro60-associated s-RNYs generated by apoptotic macrophages are released in the blood of patients, we have investigated the extracellular function of the s-RNY/Ro60 complex. Our data demonstrated that s-RNY/Ro60 complex induces caspase 3-dependent cell death and NF-*κ*B-dependent inflammation, when added to the medium of cultured monocytes/macrophages. Finally, we have shown that s-RNY function is mediated by Toll-like receptor 7 (TLR7). Indeed using chloroquine, which disrupts signaling of endosome-localized TLRs 3, 7, 8 and 9 or the more specific TLR7/9 antagonist, the phosphorothioated oligonucleotide IRS954, we blocked the effect of either intracellular or extracellular s-RNYs. These results position s-RNYs as relevant novel functional molecules that impacts on macrophage physiopathology, indicating their potential role as mediators of inflammatory diseases, such as atherosclerosis.

Macrophage apoptosis is a tightly regulated mechanism to control tissue homeostasis, resolution of inflammation^1, 2^ and innate immune response against microbial infection.^3^ Induction of macrophage cell death can be initiated by a variety of signals and stress factors, including bacterial toxins, DNA damage, oxidant stress, endoplasmic reticulum stressors, cytokines, activation of Fas death pathway, oxidized LDL (oxLDL) and saturated fatty acids.^3, 4^ Its physiopathological importance is highlighted by the fact that apoptosis in macrophages is involved in many inflammatory diseases, such as atheroscleros is^4^ and Crohn's disease.^5^ In particular, in the pathogenesis of atherosclerosis, both macrophage apoptosis and the inflammatory response have major roles from early to advanced phases of lesion formation.^6^ In early phases, macrophages engulf lipoproteins and lipids, and become the so-called foam cells, which secrete inflammatory cytokines and undergo apoptosis. Rapid efferocytic clearance of the apoptotic foam cells leads to suppression of the inflammatory response ultimately retarding lesion progression.^7^ However, in advanced lesions, macrophage apoptosis is not properly coupled with phagocytic clearance leading to the upregulation of inflammation and to necrotic plaque formation.^8^ This may ultimately lead to clinical events, such as heart attack or stroke.^6^

In addition to microRNAs, piwi-interacting RNAs (piRNAs) and siRNAs, recent analyses from high-throughput sequencing of cell cultures and tissues have revealed the existence of many other classes of small RNAs, including those from sno-RNAs and tRNAs^9^ or from Alu repeat elements,^10^ indicating that cells may generate a wide range of regulatory small RNAs with a broad variety of processing mechanisms and functions. Using small RNA high-throughput sequencing, we recently demonstrated that the ~110 nucleotides (nt) long Ro-associated Y RNAs (also called RNYs or YRNAs), are processed into small sequences in apoptotic and lipid-laden macrophages.^1, 11^ We found that the RNY-derived small RNAs vary in length from ~24 to ~34 nt and map at the end of the stem regions of the RNYs. We have referred to these small RNAs as s-RNYs. RNY genes account for four copies in human (RNY1, 3, 4 and 5) and two in mouse genome (RNY1 and 3), and their sequence is well conserved among vertebrates.^12^ The expression of s-RNYs is significantly upregulated in mouse models for atherosclerosis (namely the *ApoE*^*−/−*^ and *Ldlr*^*−/−*^ mice) and in the serum of 263 patients with coronary artery disease (CAD) compared with 514 age-matched controls.^1, 11^ Biostatistical analysis positioned s-RNYs as relevant novel independent diagnostic biomarkers for CAD and associated it with atherosclerosis burden.^1, 11^

In this report, we investigated the role of s-RNYs in human and mouse monocytes/macrophages. By either inducing or inhibiting the expression, we have shown that s-RNYs activate both caspase-dependent cell death and NF-*κ*B-dependent inflammation in cultured monocytes/macrophages. This phenotype was rescued by treating the monocytes/macrophages with chloroquine and IRS954 (specific inhibitor of Toll-like receptor 7 (TLR7)), indicating that s-RNYs activate TLR7. Finally, the isolated s-RNY/Ro60 complex by affinity purification also promoted TLR7-dependent apoptosis and inflammation when added in the medium of cultured monocytes/macrophages. This effect was rescued by s-RNY antisense oligonucleotides and by TLR7 inhibitors. Overall, these data indicate that s-RNYs are intrinsic components of the machinery regulating lipid-laden macrophage phenotype and function as mediators of inflammation/apoptosis. In atherosclerosis, we speculate that s-RNYs promote the progression of the disease. Endothelial cells, which do not express TLR7 or s-RNYs when stimulated with pro-atherogenic agents,^1^ are not activated by s-RNYs, confining the extracellular role of s-RNYs to immune cells.

## Results

### Pro-atherogenic stimuli induce the expression of RNY-derived small RNAs in monocytes/macrophages

To explore the potential involvement of s-RNYs in regulating macrophage foam cells, we first analyzed the expression pattern of s-RNYs in oxLDL-treated or palmitic acid (PA)-treated macrophages in a time course experiment. Either oxLDL or PA treatment promotes both apoptosis and inflammatory response by regulating signaling pathways downstream TLRs activation, leading to the upregulation of NF*-κ*B-dependent inflammation and caspase-dependent cell death.^13, 14, 15, 16^

**Figure 1:**
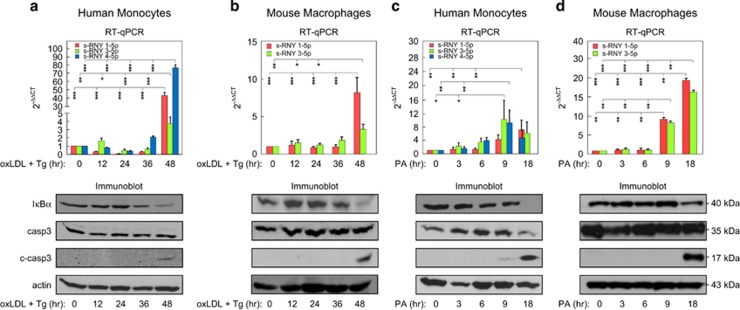
Atherogenic stimuli induce s-RNY expression in monocytes/macrophages, which is timely correlated with inflammatory and cell death responses. Quantitative RT-PCR detecting the indicated s-RNYs at the indicated time in either human THP1 monocytes or BMDMs stimulated with either 100 *μ*g/ml of oxLDL in combination with 0.25 μM of Tg (upper panels of **a** and **b**, respectively) or with 0.25 mM of PA in complex with BSA (upper panels of **c** and **d**, respectively). The data were normalized by U2 snRNA and presented as mean and S.D. (*n*=4). Each condition was subjected to immunoblot with the indicated antibodies (lower panels) to analyze the time correlation between the s-RNY induction and the inflammatory or cell death responses. One-way ANOVA with *post-hoc* Tukey test: *P<0.05; **P<0.01; ***P<0.001

In accordance with previous reports,^16, 17^ we found apoptosis and inflammation activation in monocytes/macrophages treated with pro-atherogenic stimuli, such as PA or oxLDL in combination with thapsigargin (Tg) (Figure 1). In particular, our data revealed that either oxLDL or PA treatment of human monocytes or mouse macrophages activated caspase-dependent apoptosis, which depends on the cleavage of caspase 3, after 48 and 9 h, respectively (Figure 1). NF-*κ*B, which depends on I*κ*B*α* degradation, was also activated after 48 and 9 h, respectively. Importantly, as shown in Figure 1, s-RNY expression was also induced after 48 and 9 h of oxLDL or PA treatment, respectively. Small RNA derived from the RNY5 was not measured by stem-loop RT-qPCR because only the 3' fragment is generated from this RNA in lipid-laden macrophages.^1^ These data indicate a timing correlation between s-RNY induction and cell signaling activation, inferring a possible role of s-RNYs in regulating both NF-*κ*B-dependent inflammation and caspase-dependent cell death.

### s-RNYs regulate apoptosis and inflammatory response in lipid-laden monocytes/macrophages

**Figure 2:**
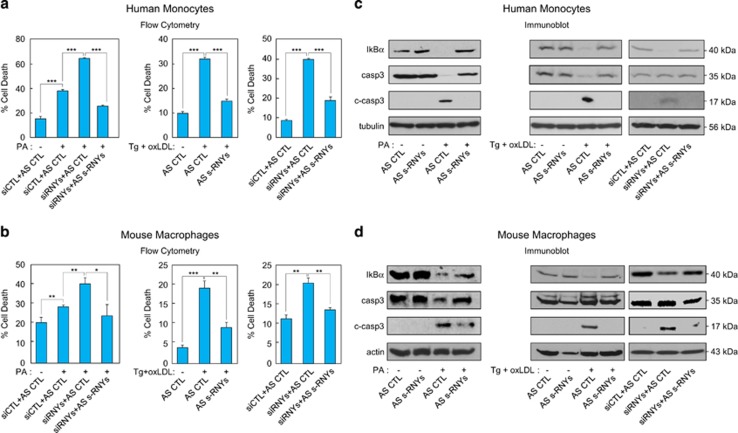
s-RNYs promote apoptosis and inflammation in lipid-laden macrophages. Cell death data by flow cytometry from human THP1 cells (**a**) or mouse BMDMs (**b**) transfected with a cocktail of siRNAs against the terminal loop of all RNYs (siRNYs) to induce s-RNY maturation, a cocktail of 2'-OMe-RNA antisense oligonucleotides (AS) to all s-RNYs expressed, or control. Cells were stimulated with 0.25 mM of PA for 18 h (left panel), 0.25 *μ*M Tg in combination with 100 *μ*g/ml of oxLDL for 48 h (middle panel) or left unstimulated (right panel). Percentage of apoptotic cells was determined by staining with annexin V-FITC. Data are presented as mean and S.D. (*n*=3). Immunoblot analysis of I*κ*B*α*, actin, total caspase 3 (casp3) and its cleaved form (c-casp3) in THP1 cells (**c**) or BMDMs (**d**) transfected with either a cocktail of AS to all s-RNYs or control. Cells were stimulated with 0.25 mM of PA for 18 h (left panel), 0.25 *μ*M Tg in combination with 100 *μ*g/ml of oxLDL for 48 h (middle panel) or left unstimulated (right panel). Student's *t*-test: *P<0.05; **P<0.01; ***P<0.001

We evaluated the effects of s-RNYs on macrophage apoptosis using flow cytometry. As siRNA against the terminal loop of RNY generates s-RNYs (Supplementary Figures S1a and c) associated with Ro60 (ref.^18^) mimicking the induction of the processing that occurs in lipid-laden macrophages and apoptotic monocytes/macrophages, we used this strategy to induce s-RNY expression. We found that inducing s-RNY expression caused an upregulation of the apoptosis mediated by PA treatment of monocytes/macrophages (left panels of Figures 2a and b). We rescued the apoptosis upregulation by co-transfecting the 2'-OMe-RNA antisense oligonucleotides to s-RNYs (AS s-RNYs) (Supplementary Figure S1b), indicating that this effect was specific of s-RNYs. Similarly, we found a downregulation of the apoptosis when we knocked down endogenous s-RNY expression in monocytes/macrophages treated with oxLDL/Tg (middle panels of Figures 2a and b). Notably, by promoting s-RNY maturation we induced the apoptosis in untreated monocytes/macrophages, which was also rescued by co-transfecting the 2'-OMe-RNA antisense oligonucleotides to s-RNYs (right panels of Figures 2a and b). These results indicate that atherogenic stimuli-induced s-RNYs are an intrinsic component of the machinery regulating apoptosis in lipid-laden monocytes/macrophages.

This conclusion was confirmed by western blot. Indeed, as shown in Figures 2c and d, caspase 3 was activated by inducing s-RNY expression, while we observed a significant reduction upon s-RNY knockdown in PA- or oxLDL-treated monocytes/macrophages, or in s-RNY-induced apoptosis. These data indicate that s-RNYs promote caspase-dependent apoptosis in monocytes/macrophages. Moreover, s-RNYs were also able to activate NF-*κ*B pathway in either PA- or oxLDL-treated or unstimulated monocytes/macrophages, as we demonstrated by checking the expression levels of I*κ*B*α* (Figures 2c and d). Moreover, we tested the effect of inducing s-RNY maturation on the expression regulation of death cytokines and nitric oxide activated by NF-*κ*B pathway in bone marrow-derived macrophages (BMDMs) treated with the general macrophage activator lipopolysaccharide (LPS). As shown in Supplementary Figure S2, s-RNYs enhanced a significant upregulation of the mRNAs encoding for NOS2A, interleukin (IL)-6, IL-12b and IL-1b. Therefore these data indicate that s-RNYs activate caspase-dependent cell death and NF-*κ*B-dependent inflammation in lipid-laden monocytes/macrophages.

### Extracellular s-RNY/Ro60 complex promotes apoptosis and inflammation in monocytes/macrophages

**Figure 3:**
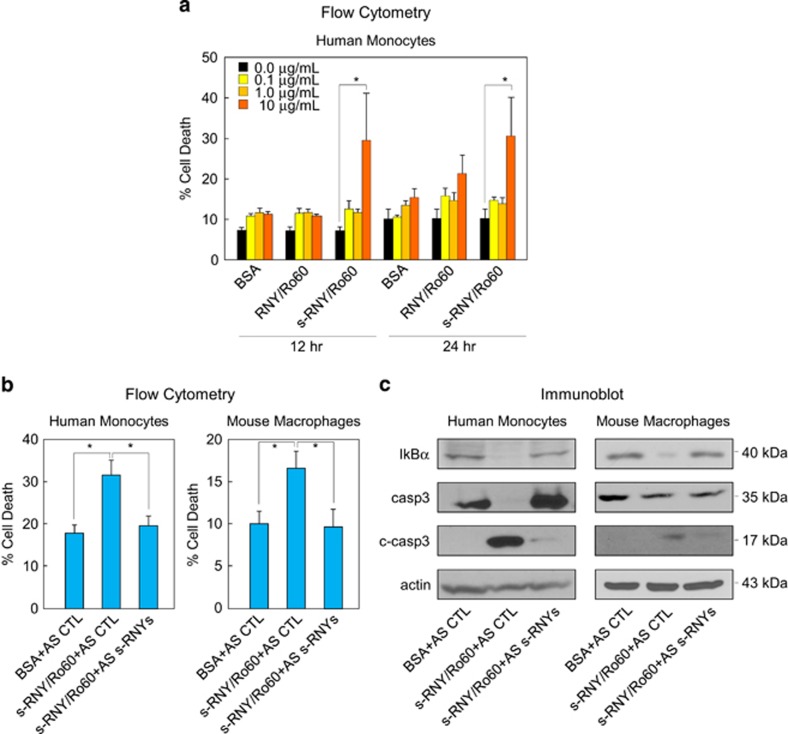
Extracellular s-RNYs promote apoptosis and inflammation in lipid-laden macrophages. (**a**) Time and dose response of cell death by flow cytometry from human THP1 cells incubated with the immunopurified complex of s-RNY/Ro60 or RNY/Ro60. Incubation with BSA was used as control. Data are presented as mean and S.D. (*n*=5). One-way ANOVA with *post-hoc* Tukey test: *P<0.05. (**b**) Flow cytometry from human THP1 cells (left panel) or mouse BMDMs (right panel) incubated with 10 *μ*g/ml of either the immunopurified complex of s-RNY/Ro60 or BSA, as control. s-RNY/Ro60 complex was previously incubated for 16 h in rotation at 4 °C with either 40 nM of 2'-OMe-RNA AS to s-RNYs or control. Data are presented as mean and S.D. (*n*=5). Student's *t*-test: *P<0.05. (**c**) Immunoblot analysis of I*κ*B*α*, actin, total caspase 3 (casp3) and its cleaved form (c-casp3) in THP1 cells (left panel) or BMDMs (right panel) incubated s-RNY/Ro60 complex previously pretreated with either AS to s-RNYs or control

There is evidence that s-RNYs are associated with Ro60, and that this association is most likely responsible for the stability of s-RNYs.^1, 19^ Given that s-RNYs have been found upregulated in the serum of patients with CAD compared with control,^1^ we investigated whether the extracellular s-RNY/Ro60 complex also contributes to the apoptotic and inflammatory phenotype of lipid-laden macrophages. Briefly, we immunopurified the Ro60 complex from staurosporine (STS)-treated Hek-293T cells and control, reasoning that Ro60 is mainly associated with RNY in control cells, while it is mainly associated with s-RNYs in STS-treated cells (Supplementary Figure S3a and b). Either RNY/Ro60 or s-RNY/Ro60 complexes were added into the medium of cultured monocytes at different concentration and time. As shown in Figure 3a, only s-RNY/Ro60 complex at the minimal dose of 10 *μ*g/ml induced cell death after at least 12 h of incubation. Importantly, to determine a specific pro-apoptotic effect of the s-RNYs, we incubated the s-RNY/Ro60 complex with the 2'-OMe-RNA antisense oligonucleotides to s-RNYs. As shown in Figures 3b and c, in human monocytes or mouse primary macrophages the s-RNY/Ro60 complex-induced apoptosis was blocked by using s-RNY antisense oligonucleotides. As we have shown for the intracellular, also the extracellular s-RNYs promote caspase 3 and NF-*κ*B activation by inducing cleaved caspase 3 and inhibiting I*κ*B*α* expression levels (Figure 3c). However, naked synthetized s-RNYs added in the medium was not able to activate monocytes/macrophages (Supplementary Figure S4a), while they enhanced apoptosis and inflammation when transfected (Supplementary Figure S4b). These data may indicate that s-RNYs need to be complexed with Ro60 to get internalized by the cells and activate them. Moreover, RNY/Ro60 complex does not have any effect (Figure 3a), indicating that the pro-apoptotic and pro-inflammatory effect is only specific to the s-RNYs. Overall, these data indicate that extracellular s-RNYs but not the long form RNY induce caspase-dependent cell death and NF-*κ*B-dependent inflammation in monocytes/macrophages.

### s-RNYs activate TLR7 to induce apoptosis and inflammation in monocytes/macrophages

**Figure 4:**
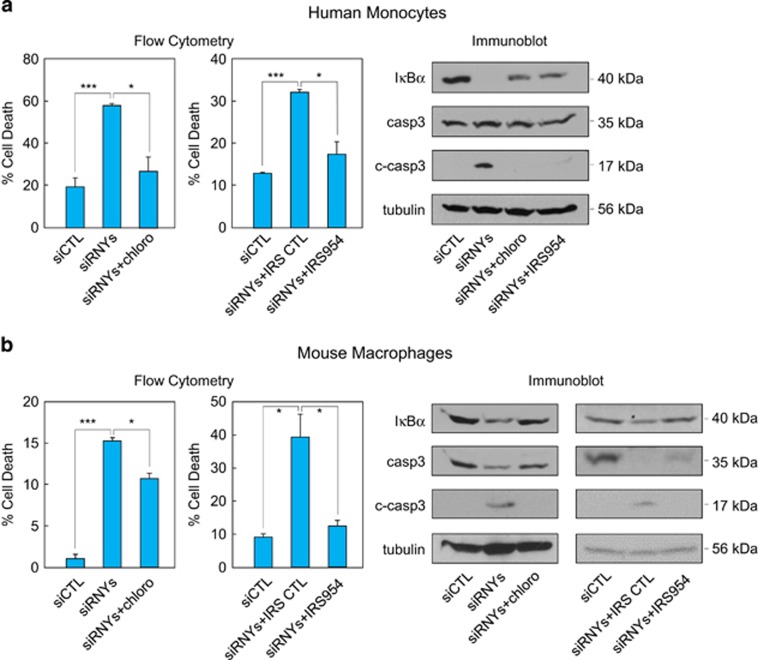
Intracellular s-RNYs activate TLR7 to promote apoptosis and inflammation in monocytes/macrophages. Cell death data by flow cytometry from human THP1 cells (**a** – left panels) or mouse BMDMs (**b** – left panels) transfected with a cocktail of siRNAs against the terminal loop of all RNYs (siRNYs) to induce s-RNY maturation or control (siCTL). Cells were incubated with 50 mM of chloroquine for 48 h (left panels) or with the TLR7/9 specific inhibitor IRS954 at 10 *μ*g/ml of concentration for 48 h (middle panels). Percentage of apoptotic cells was determined by staining with annexin V-FITC. Data are presented as mean and S.D. (*n*=3). The same experimental conditions were analyzed by immunoblot of I*κ*B*α*, actin, total caspase 3 (casp3) and its cleaved form (c-casp3) in THP1 cells (**a** – right panel) or BMDMs (**b** – right panels). Student's *t*-test: *P<0.05; ***P<0.001

**Figure 5:**
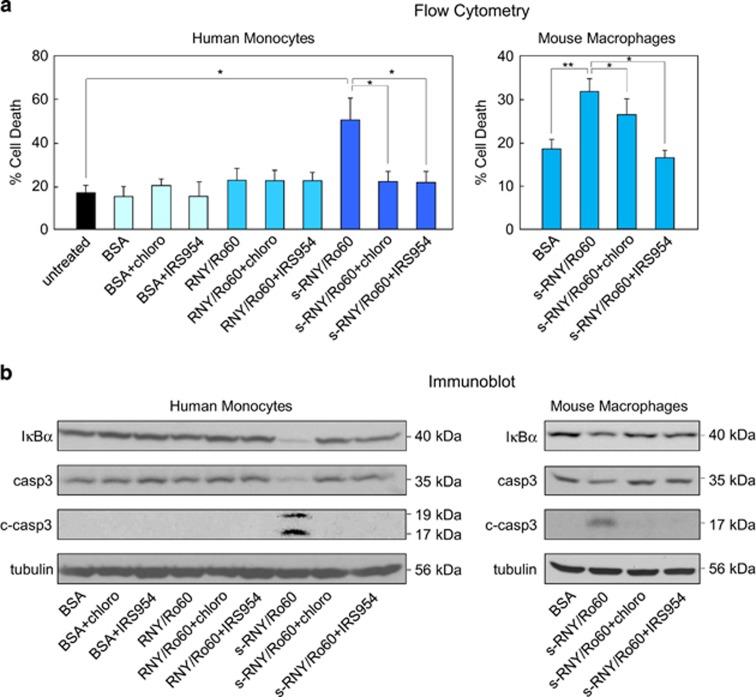
Extracellular s-RNYs activate TLR7 to promote apoptosis and inflammation in monocytes/macrophages. Cell death data by flow cytometry from human THP1 cells (**a** – left panel) or mouse BMDMs (**a** – right panel) incubated with 10 *μ*g/ml of the immunopurified complex of s-RNY/Ro60, RNY/Ro60 or BSA. Cells were incubated with 50 mM of chloroquine for 24 h or with IRS954 at 10 *μ*g/ml of concentration for 24 h, as indicated. Percentage of apoptotic cells was determined by staining with annexin V-FITC. Data are presented as mean and S.D. (*n*=6 for BMDMs and 4 for THP1 cells). The same experimental conditions were analyzed by immunoblot of I*κ*B*α*, actin, total caspase 3 (casp3) and its cleaved form (c-casp3) in THP1 cells (**b** – left panel) or BMDMs (**b** – right panel). Student's *t*-test: *P<0.05; **P<0.01

As anti-Ro60 single-stranded RNA-containing immunocomplex stimulates inflammation through TLR7/8,^20, 21^ we have investigated whether the s-RNY-induced apoptosis and inflammation in monocytes/macrophages is also mediated by the activation of intracellular TLRs. We therefore evaluated whether the chloroquine, a molecular agent that disrupts signaling of endosome-localized TLRs 3, 7, 8 and 9 or the more specific TLR7/9 antagonist, the phosphorothioated oligonucleotide IRS954,^22^ were able to inhibit the pro-apoptotic and pro-inflammatory effects of s-RNYs. As TLR9 is supposed to recognize only DNA, in our case the use of oligonucleotide IRS954 was meant to check whether s-RNYs specifically activate TLR7, which recognizes single-stranded RNA. Importantly, as shown in Figures 4a and b, flow cytometry and western blot analyses indicate that intracellular s-RNY-dependent activation of monocytes/macrophages was inhibited by chloroquine or IRS954. In particular, chloroquine or IRS954 blocked the s-RNY induction of caspase 3-dependent apoptosis and the I*κ*B*α* downregulation-dependent inflammation. Similar results were obtained for the extracellular s-RNYs (Figures 5a and b). Overall, these data indicate that TLR7 activation is downstream the s-RNY mode of action and mediates the s-RNY-dependent apoptotic and inflammatory response in monocytes/macrophages.

Taken together, our data suggest that s-RNY is an intrinsic component of the machinery regulating lipid-laden macrophage phenotype and function as mediator of inflammation/apoptosis. Endothelial cells, which do not express TLR7 or s-RNYs when stimulated with oxLDL/Tg,^1^ are not activated by extracellular s-RNYs (Supplementary Figure S5), confining to immune cells the extracellular role of s-RNYs during atherosclerosis pathogenesis.

## Discussion

Recently, we have uncovered for the first time a previously unsuspected aspect of macrophage activation whereby atherogenic lipids and lipoproteins induce the processing of the RNA-Polymerase III-driven non-coding RNYs into small RNAs, the s-RNYs.^1^ RNYs are characterized by extensive base pairing of the 5' and 3' regions and by the association with the proteins Ro60 and La/SSB, which are often targeted by the immune system in several autoimmune diseases.^23^ Although, the role of RNYs *per se* is still poorly understood, s-RNYs have been previously described in T cells stimulated with anti-FAS antibody,^23^ in germ cells, and in acute lymphoblastic leukemia.^24, 25^ In addition, we found that the expression of s-RNYs is significantly upregulated in aortic arches and blood of two mouse models for atherosclerosis, the *ApoE*^*−/−*^ and *Ldlr*^*−/−*^ mouse lines. Moreover, we also measured the s-RNY expression in 263 sera of patients with CAD and 514 sera from age-matched control individuals.^1^ Biostatistical analysis positioned s-RNYs as relevant novel independent diagnostic biomarkers for CAD.^1^ The findings we are presenting here reveal for the first time that both intracellular or extracellular s-RNYs increase inflammation and cell death in lipid-laden macrophages by activating TLR7. RNY, from which s-RNY derived, is not able to activate macrophages, suggesting that only s-RNY can be recognized by TLR7. In concordance to this mechanism, upon s-RNY induction we observed a significant upregulation of cleaved caspase 3 and downregulation of I*κ*B*α*, which activate apoptosis and inflammation, respectively. In fact, I*κ*B protein family (mainly I*κ*B*α*) inhibits the DNA binding activity of NF-*κ*B transcription factors, which have an important role in inflammation and in cell survival/apoptosis balance in macrophages.^26^ Noteworthy, we found this novel function of s-RNYs is conserved in human and mouse monocytes/macrophages. Interestingly, also the sequence of s-RNYs and the structure of the precursor RNYs are well conserved between human and mouse.^12^ Thus, a conserved pathway of s-RNY maturation and function may provide at least some of the evolutionary pressure to maintain such a mechanism in mammals.

The presence of small RNAs in the extracellular environment ignited the hypothesis that cells selectively release small RNAs to mediate cell–cell signaling via paracrine or endocrine routes.^27^ In particular, extracellular miRNAs have been found to associate to Ago proteins and form stable ribonucleoprotein complexes or entrapped to extracellular vesicles, such as apoptotic bodies and exosomes. Only miRNAs circulating in extracellular vesicles can be transferred to recipient cells and alter gene expression programmes.^27^ Along with others, we have found that circulating levels of s-RNYs are dysregulated in cardiovascular disorder ^1, 28^ and in aging.^29^ Here, we have demonstrated that as miRNAs, also extracellular Ro60-associated s-RNYs stimulate cell death and inflammation of recipient immune cells. Importantly, extracellular synthetic s-RNYs cannot activate monocytes/macrophages, suggesting that the association between s-RNY and Ro60 would permit the internalization of s-RNY into the cells.

There is evidence that most regulatory small RNA families target multiple genes that have related functions, and thereby exert strong physiopathological effects.^30^ s-RNYs are good examples in lipid-laden macrophages, because they activate caspase 3 and NF-*κ*B pathways driving cell death and inflammation and, doing so, effectively reinforcing pro-atherogenic networks. Therefore, in addition to miRNAs, such as miR-155,^31^ s-RNYs represent a major group of small RNAs that regulate immune response in the pathogenesis of atherosclerosis. In accordance with this conclusion, our previous study unraveled a significant increase of s-RNY expression during atherosclerosis development and in the serum of patients with stable CAD, indicating that s-RNY can be potentially used as therapeutic targets for atherosclerosis.

**Figure 6:**
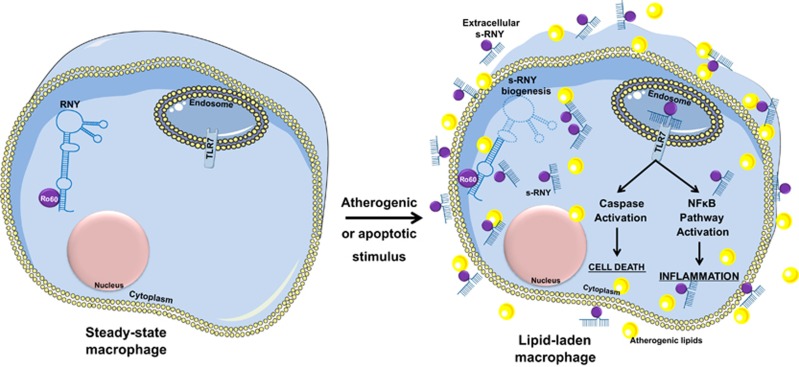
A model for s-RNY mode of action in lipid-laden monocytes/macrophages that exerts signaling pathway effects enhancing cell death and inflammation

In conclusion, our results uncover a strategy by which atherogenic lipids and lipoproteins induce the biogenesis of s-RNYs in macrophages to enhance inflammation and cell death by activating TLR7 (Figure 6). We speculate that s-RNYs promote the progression of the disease, as extracellular s-RNYs activate the apoptotic and inflammatory response to unstimulated monocytes/macrophages. Altogether, this study contributes to the diversity of the regulatory small RNA profile that impacts on the physiopathology of lipid-laden macrophages and atherosclerosis development. As inflammation and apoptosis in macrophages are tightly regulated mechanisms and their physiopathological importance is highlighted by the fact that its dysregulation is involved in many human diseases, such as atherosclerosis,^7^ Crohn's disease^5^ and microbial infections,^3^ it is tempting to speculate that s-RNYs may have a key role in regulating diverse aspects of development, homeostasis and diseases.

## Materials and Methods

### Reagents

LPS, Tg, STS, bovine serum albumin (BSA) and PA were purchased from Sigma (Saint-Quentin Fallavier, France). oxLDL was purchased from Clinisciences (Nanterre, France). siRNA control was from Dharmacon (GEHealthCare Europe, Villebon Sur Yvette, France), whereas control 2'-OMe-RNA antisense oligonucleotide was from Qiagen France Sas (Courtaboeuf Cedex, France). PA was solubilized in sterile water at 70 °C for 30 min, then 1% of 1 N NaOH was added each 5 min for 10 min, vortexing each time. Afterward, the solubilized PA was mixed with 1% of fatty acid-free BSA in Dulbecco's modified Eagle's medium (DMEM) at the final 2 mM. For PA treatment of cells, we used as control 1% of fatty acid-free BSA-treated cells in FBS-free DMEM.

### Primary macrophages

Bone marrow cells were collected from femurs and tibias of 10-week-old male C57BL/6 J mice by flushing with sterile medium as previously described.^32^ To differentiate into macrophages, bone marrow cells (10^6^) were plated in 10-cm plates in 7 ml of BMDM medium (DMEM supplemented with 20% low-endotoxin fetal bovine serum, 30% L929-cell conditioned medium, 1% l-glutamine, 1% Pen/Strep, 0.5% Na pyruvate and 0.1% *β*-mercaptoethanol), and fed with 2.5 ml of fresh medium every 2 days for 7 days.

### Cell transfection and immunoblotting

BMDMs and the monocyte cell line THP1 (ATCC/LGC standards, Molsheim Cedex, France) were transiently transfected for 48 h with Lipofectamine 2000 (Invitrogen/Life Technologies, Villebon Sur Yvette, France) according to the manufacturer's instructions. Either siRNAs or chemically synthetized s-RNYs (Eurogentec SA, Angers, France) were transfected at the final concentration of 24 nM, whereas 2'-OMe-RNA antisense oligonucleotides to s-RNYs (Eurogentec) were transfected at the final concentration of 100 nM. Sequence of siRNAs and antisense oligonucleotides are indicated below.

### Immunopurification of s-RNY/Ro60 complex

In all, 10-cm dishes of unstimulated or STS-stimulated Hek-293T cells were irradiated once at 400mJ/cm^2^ with 254nm UVC light. Afterward, cells were lysate and incubated 16 h at 4 °C with rotation with anti-Ro60 antibody (sc-100844 – Santa Cruz Biotechnology Inc., Heidelberg, Germany). The anti-Ro60 antibody was previously covalently bound to protein G/magnetic beads by 25 mM of dimethypimelimidate dihydrochloride (Sigma, D-8388) dissolved in 0.2 M triethanolamine according to the manufacturer's instruction. Immunoprecipitation was washed four times and the s-RNY(RNY)/Ro60 complex was eluted with 30*μ*l of 0.1 M glycin-HCl (pH 2.5) and then neutralized with 3*μ*l of 1 M Tris at pH 7.5. When indicated, the s-RNY/Ro60 complex was incubated for 16 h in rotation at 4 °C with either 40 nM of 2'-OMe-RNA antisense oligonucleotides against the s-RNYs (human or mouse) or control.

### Apoptosis assay

Apoptosis was assayed using flow cytometry in BMDMs or THP1 cells by staining with FITC-conjugated annexin V and Dapi as previously described.^33^ The number of annexin V-positive cells was counted and expressed as a percent of the total number of cells.

### Northern blot

Northern blot from RNA immunoprecipitation was performed as previously described.^34^ RNA was isolated from cells using Trizol (Invitrogen), resolved on 10% polyacrylamide-urea gels and electroblotted onto HyBond N+ membranes. Membranes were hybridized overnight with radiolabeled DNA antisense oligonucleotides to s-RNYs in ExpressHyb solution (Clontech/Ozyme Saint-Quentin En Yvelines, France). After hybridization, membranes were washed three times with 2X SSC and 0.05% SDS, two times with 0.1X SSC and 0.1% SDS, exposed overnight onto a film. Northern blot probe is detailed in Supplementary Table S1.

### mRNA and s-RNY expression analysis

RNA expression by quantitative RT-PCR was carried out by using standard procedures. Briefly, for mRNA, total RNA was isolated from cells using Trizol and cDNA was synthesized with a random hexonucleotides using Superscript III (Invitrogen). For s-RNYs detection by quantitative RT-PCR, we used a stem-loop quantitative RT-PCR method according Repetto *et al.*^1^ This method allows the detection of the s-RNYs derived from only the 5' end of the precursor by quantitative RT-PCR analysis, namely the s-RNY1-5p and s-RNY3-5p from mouse and s-RNY1-5p, s-RNY3-5p, and s-RNY4-5p from human. Quantitative RT-PCRs using Sybr Green (Invitrogen) were performed on a StepONE system (Applied Biosystem/Life Technologies SAS, Villebon Sur Yvette, France). Expression was considered undetectable with Ct value ≥40. Target small RNA expression value was normalized with two reference endogenous genes: U2 or U6 snRNAs. The relative expression level was then further normalized by the 2^−ΔΔCt^ method. Student's *t*-test was performed to assess statistical significance. The primer sequences are detailed in Supplementary Table S1.

### Antibodies for western blot

Goat anti-actin (I-19) and I*κ*B*α* (H-4) and mouse anti-Ro60 (AA3) antibodies were purchased from SantaCruz. Rabbit anti-cleaved caspase 3 (Asp175) and rabbit anti-caspase 3 (8G10) antibodies were purchased from Cell Signaling/Ozyme Saint-Quentin En Yvelines (France).

### siRNAs and modified-RNA oligonucleotides

Custom siRNAs or modified-RNA oligonucleotides were synthesized by Eurogentec. Regular oligonucleotides were synthesized by Sigma.

- Mouse/human siRNA against RNY1 terminal loop 5′-CAGUCAGUUACAGAUUGAA-3′.
- Mouse/human siRNA against RNY3 terminal loop 5′-CAACCAGUUACAGAUUUCU-3′.
- Mouse/human 2'-OMe-RNA antisense to s-RNY1-5p 5′-UUGAGAUAACUCACUACCUUCGGACCAGCC-3′.
- Mouse/human 2'-OMe-RNA antisense to s-RNY1-3p 5′-AAGACUAGUCAAGUGCAGUAGUGAGAAG-3′.
- Mouse 2'-OMe-RNA antisense to s-RNY3-5p 5′-UAAACACCACUACUCUCGGACCAACC-3′.
- Human 2'-OMe-RNA antisense to s-RNY3-5p 5′-UAAACACCACUGCACUCGGACCA-3′.
- Human 2'-OMe-RNA antisense to s-RNY3-3p 5′-AAGGCUAGUCAAGUGAAGCAGUGGGAG-3′.
- Mouse 2'-OMe-RNA antisense to s-RNY3-3p 5′-AAGGCUGGUCAAGUGAAGCAGUGGGAGC-3′.
- Human siRNA against RNY4 terminal loop 5′-GUGUCACUAAAGUUGGUAU-3′.
- Human siRNA against RNY5 terminal loop 5′-AGUUGAUUUAACAUUGUCU-3′.
- Human 2'-OMe-RNA antisense to s-RNY4-5p 5′-UUCUGAUAACCCACUACCAUCGGACCAGCC-3′.
- Human 2'-OMe-RNA antisense to s-RNY4-3p 5′-AAGCCAGUCAAAUUUAGCAGUGGGGGG-3′.
- Mouse 2'-OMe-RNA antisense to s-RNY5-3p 5′-CAAGCGCGGUUGUGGGGGGA-3′.
- IRS954 5′-TGCTCCTGGAGGGGTTGT-3′.
