# Supplementary material for: RNY (YRNA)-derived small RNAs regulate cell death and inflammation in monocytes/macrophages
Source: Cell Death Dis. 2017 Jan 5;8(1):e2530–. doi: 10.1038/cddis.2016.429 (PMC5386355; doi:10.1038/cddis.2016.429)
Supplement: Supplementary Information [file cddis2016429x1.doc]

**SUPPLEMENTAL INFORMATION**

Table S1. Oligonucleotide sequences used.

|  | Forward primer | Reverse primer |
| --- | --- | --- |
| Mouse/human s-RNY1-5p (RT) |  | 5’- GTCGTATCCAGTGCAGGGTCCGAGGTATTCGCACTGGATACGACATTGAG -3’ |
| mouse s-RNY3-5p (RT) |  | 5’- GTCGTATCCAGTGCAGGGTCCGAGGTATTCGCACTGGATACGACAACACC -3’ |
| human s-RNY3-5p (RT) |  | 5’- GTCGTATCCAGTGCAGGGTCCGAGGTATTCGCACTGGATACGACAGTTGT -3’ |
| human s-RNY4-5p (RT) |  | 5’- GTCGTATCCAGTGCAGGGTCCGAGGTATTCGCACTGGATACGACAGTTCT -3’ |
| Mouse U2 snRNA(qPCR) | 5’-GAAGTAGGAGTTGGAATAGGA -3’ | 5’-ACCGTTCCTGGAGGTACTG -3’ |
| Human/Mouse U6 snRNA (qPCR) | 5’- CGCTTCGGCAGCACATATAC -3’ | 5’- AAATATGGAACGCTTCACGA -3’ |
| Human U2 snRNA(qPCR) | 5’- GAGCAGGGAGATGGAATAGGA -3’ | 5’-ACCGTTCCTGGAGGTACTG -3’ |
| Mouse NOS2A (q-PCR) | 5’- agccttgcatcctcattgg -3’ | 5’- cactctcttgcggaccatct -3’ |
| Mouse IL-6 (q-PCR) | 5’- GTTCTCTGGGAAATCGTGGA -3’ | 5’- TCCAGTTTGGTAGCATCCATC -3’ |
| Mouse IL-12b (q-PCR) | 5’- ccattttccttcttgtggagca -3’ | 5’- agacatggagtcataggctctg -3’ |
| Mouse IL-1b (q-PCR) | 5’- TGGGCCTCAAAGGAAAGAAT -3’ | 5’- CAGGCTTGTGCTCTGCTTGT -3’ |
| s-RNYs universal primer (qPCR) |  | 5’- GTGCAGGGTCCGAGGT -3’ |
| Mouse/human s-RNY1-5p (qPCR) | 5’- TGGTCCGAAGGTAGTGAGT -3’ |  |
| Mouse s-RNY3-5p (qPCR) | 5’- TTGGTCCGAGAGTAGTGGT -3’ |  |
| Human s-RNY3-5p (qPCR) | 5’- TCCGAGTGCAGTGGTGTTTA -3’ |  |
| Human s-RNY4-5p (qPCR) | 5’- GGTCCGATGGTAGTGGGTTAT -3’ |  |
| Human/mouse s-RNY1-5p (Northern blot) |  | 5’- ACTCACTACCTTCGGACCA -3’ |
| U6 snRNA (Northern blot) |  | 5’-CGTTCCAATTTTAGTATATGTGCTGCCGAAGCGAGCAC-3’ |
| Mouse s-RNY3-5p (Northern blot) |  | 5’- ACCACTACTCTCGGACCAA -3’ |
| Mouse and humans s-RNY1-3p (Northern blot) |  | 5’- AGTCAAGTGCAGTAGTGAG -3’ |
| Human s-RNY4-5p (Northern blot) |  | 5’- CCCACTACCATCGGACCAG -3’ |
| Mouse/human s-RNY1-5p (mimic) | 5’- UGGUCCGAAGGUAGUGAGU -3’ |  |
| Mouse s-RNY3-5p (chemically synthetized) | 5’- UUGGUCCGAGAGUAGUGGU -3’ |  |
| Mouse/human s-RNY1-3p (chemically synthetized) | 5’- CUUCUCACUACUGCACUUGACUAGUCUU -3’ |  |
| Mouse s-RNY3-3p (chemically synthetized) | 5’- GCUCCCACUGCUUCACUUGACCAGCCUU -3’ |  |
| human s-RNY3-5p (chemically synthetized) | 5’- GGCUGGUCCGAGUGCAGUGGUGUUUA -3’ |  |
| human s-RNY3-3p (chemically synthetized) | 5’- ACUCCCACUGCUUCACUUGACUAGCCUU -3’ |  |
| human s-RNY4-5p (chemically synthetized) | 5’- GGCUGGUCCGAUGGUAGUGGGUUAUCAGAACU -3’ |  |
| human s-RNY4-3p (chemically synthetized) | 5’- AACCCCCCACUGCUAAAUUUGACUGGCUUU -3’ |  |
| human s-RNY5-3p (chemically synthetized) | 5’- UCCCCCCACAACCGCGCUUGACUAGCUUGCU-3’ |  |

**Supplemental Figure Legends**

**Supplemental Figure 1.** siRNAs against the terminal loop sequence of RNYs determine the generation of s-RNYs. (**a**) BMDMs were transfected with either siRNAs against the terminal loops of RNY1/3 (siRNYs) or control siRNA (siCTL). After 48 hr from the transfection, total RNA was isolated and analyzed by Northern blotting. U6 snRNA was used as loading control. (**b**) Northern blot analysis detecting the indicated s-RNYs in cells transfected with 2'-OMe-RNA antisense oligonucleotides (AS) to s-RNYs or control. BMDMs were left unstimulated or stimulated with 0.25 mM of PA for 18 hr, and total RNA was isolated and analyzed by Northern blotting. U6 snRNA was used as loading control. (**c**) THP-1 cells were transfected with either siRNAs against the terminal loops of RNY1/3/4/5 (siRNYs) or control siRNA (siCTL). After 48 hr from the transfection, total RNA was isolated and analyzed by Northern blotting. U6 snRNA was used as loading control.

**Supplemental Figure 2.** Quantitative RT-PCR analysis of NOS2A, IL-6, IL-12b, and IL-1b transcripts in BMDMs transfected with siRNAs against the terminal loop of RNYs to induce s-RNY maturation, 2'-OMe-RNA antisense oligonucleotides (AS) to s-RNYs, or control. Cells were left unstimulated or stimulated with 100 ng/ml LPS for 12 hr, and total RNA was isolated and analyzed. Data were normalized by U2 snRNA and are presented as mean and s.d. (n = 4). Student’s t-test: *P<0.05; **P<0.01.

**Supplemental Figure 3.** Immunoprecipitation of the s-RNY/Ro60 complex. (**a**) Ro60 immunoprecipitation from Hek-293T cells followed by Western blot using anti-Ro60 antibody. Cells were left unstimulated or stimulated with 1 μM of STS for 12 hr. Then, cells were subjected to UV-crosslink treatment and lysate. A gel loaded with input of the immunoprecipitation and the 0.1 μg of anti-Ro60 antibody was used as control. (**b**) Ro60 immunoprecipitation from Hek-293T cells followed by Northern blot s-RNY1-5p. Cells were left unstimulated or stimulated with 1 μM of STS for 12 hr and lysate. IgG was used as control.

**Supplemental Figure 4.** (**a**) Representative diagrams of cell death rate in THP-1 cells determined by flow cytometry. Cells were either incubated with 40 nM s-RNYs added in the medium for 24 hr or scramble RNA oligonucleotide sequences, as control. (**b**) Flow cytometry (left panel) or immunobloting (right panel) of the indicated antibodies of mouse BMDMs. Cells were transfected with 24 nM of either chemically synthetized s-RNYs or scramble single stranded RNA controls. For flow cytometry analysis, data are presented as mean and s.d. (n = 3). Student’s t-test: **P<0.01.

**Supplemental Figure 5.** Representative diagrams of cell death rate in HUVEC endothelial cells determined by flow cytometry (upper panels). Diagram merging 3 independent experiments (lower panel). Cells were either incubated with 10 μg/ml of the immunopurified complex of s-RNY/Ro60 or BSA, as control. Student’s t-test: ns - not significant
