# Supplementary figures and images for: RNY (YRNA)-derived small RNAs regulate cell death and inflammation in monocytes/macrophages
Source: Cell Death Dis. 2017 Jan 5;8(1):e2530–. doi: 10.1038/cddis.2016.429 (PMC5386355; doi:10.1038/cddis.2016.429)

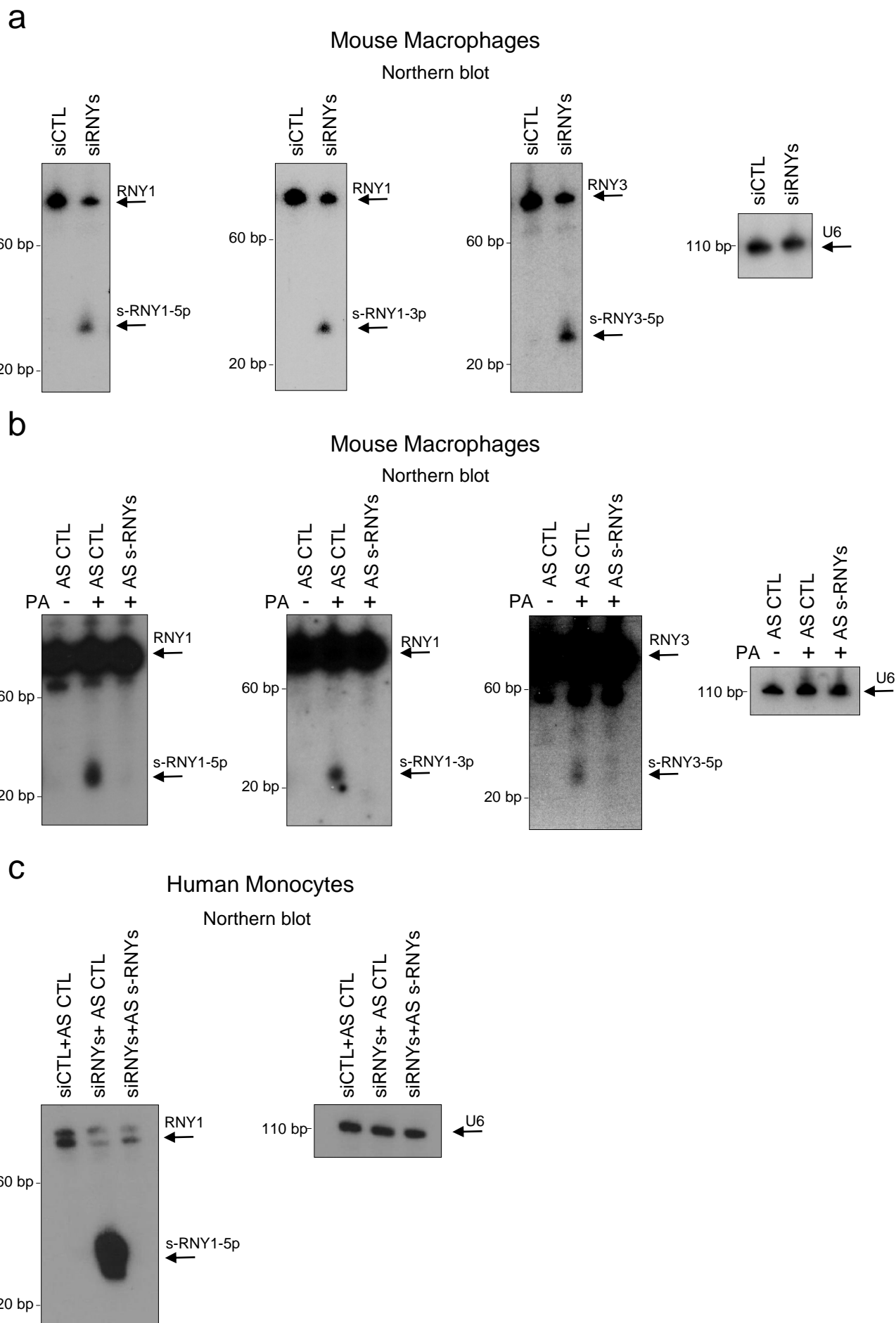

Supplement: Supplementary Figure 1 [file cddis2016429x2.pdf]

Mouse Macrophages

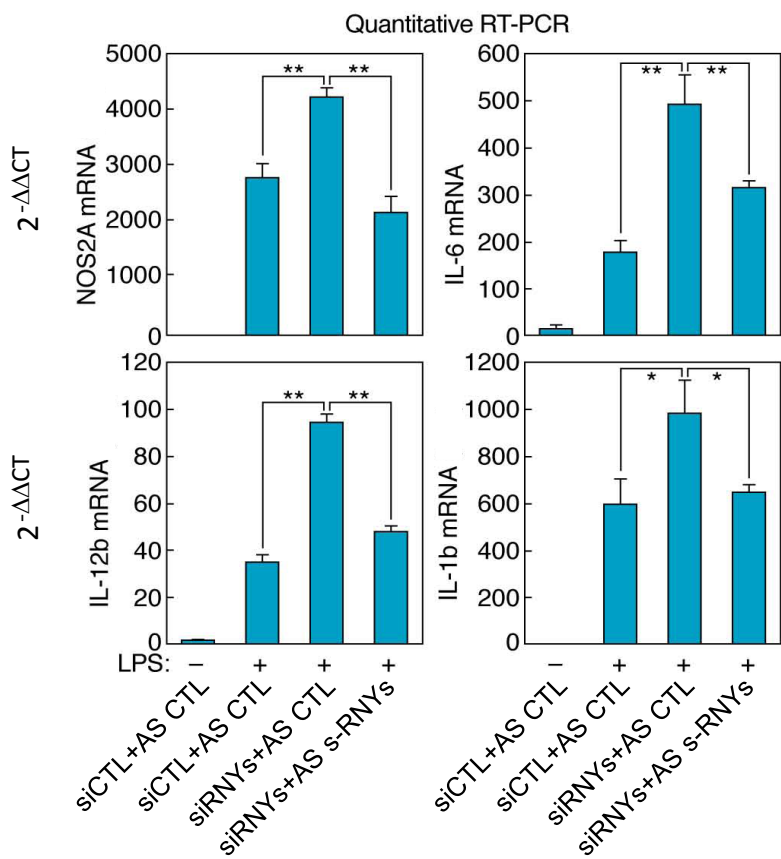

Supplement: Supplementary Figure 2 [file cddis2016429x3.pdf]

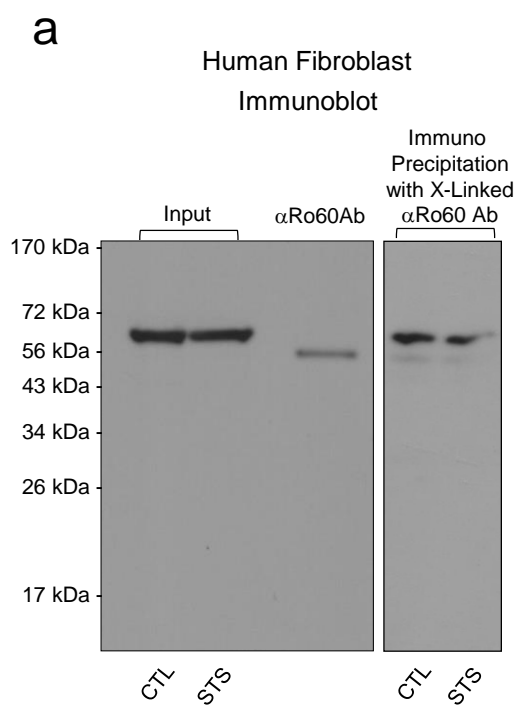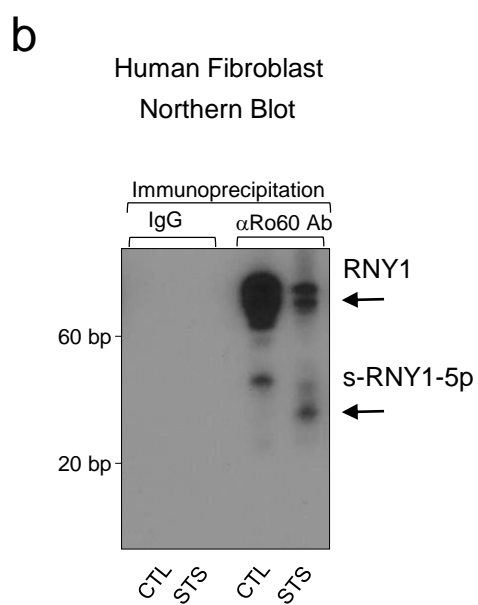

Supplement: Supplementary Figure 3 [file cddis2016429x4.pdf]

a

Flow Cytometry  
Human Monocytes

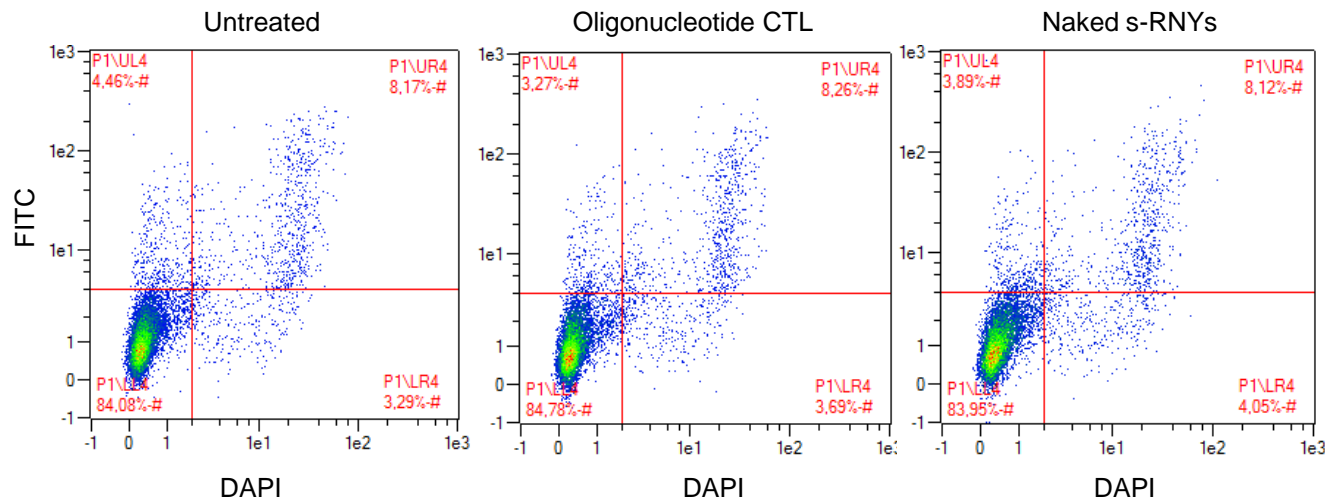

b

Mouse Macrophages

Flow Cytometry

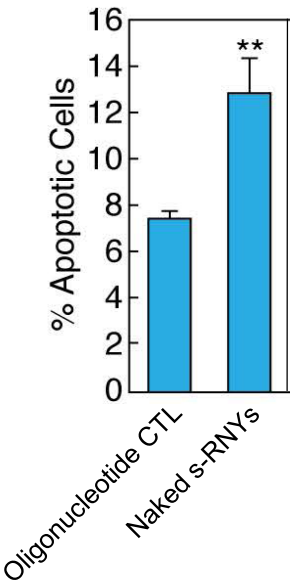

Immunoblot

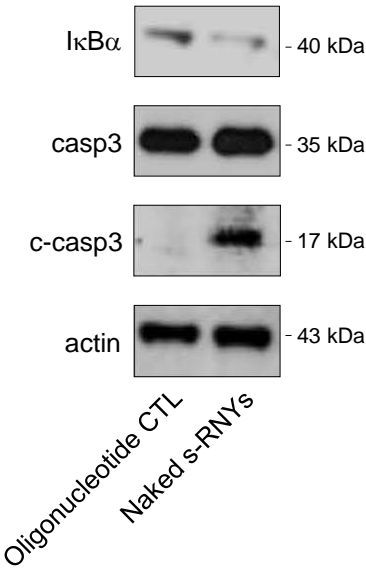

Supplement: Supplementary Figure 4 [file cddis2016429x5.pdf]
