# Supplementary material for: RNY (YRNA)-derived small RNAs regulate cell death and inflammation in monocytes/macrophages
Source: Cell Death Dis. 2017 Jan 5;8(1):e2530–. doi: 10.1038/cddis.2016.429 (PMC5386355; doi:10.1038/cddis.2016.429)
Supplement: Supplementary Figure 5 [file cddis2016429x6.pdf]

Flow Cytometry

Human Endothelial cells

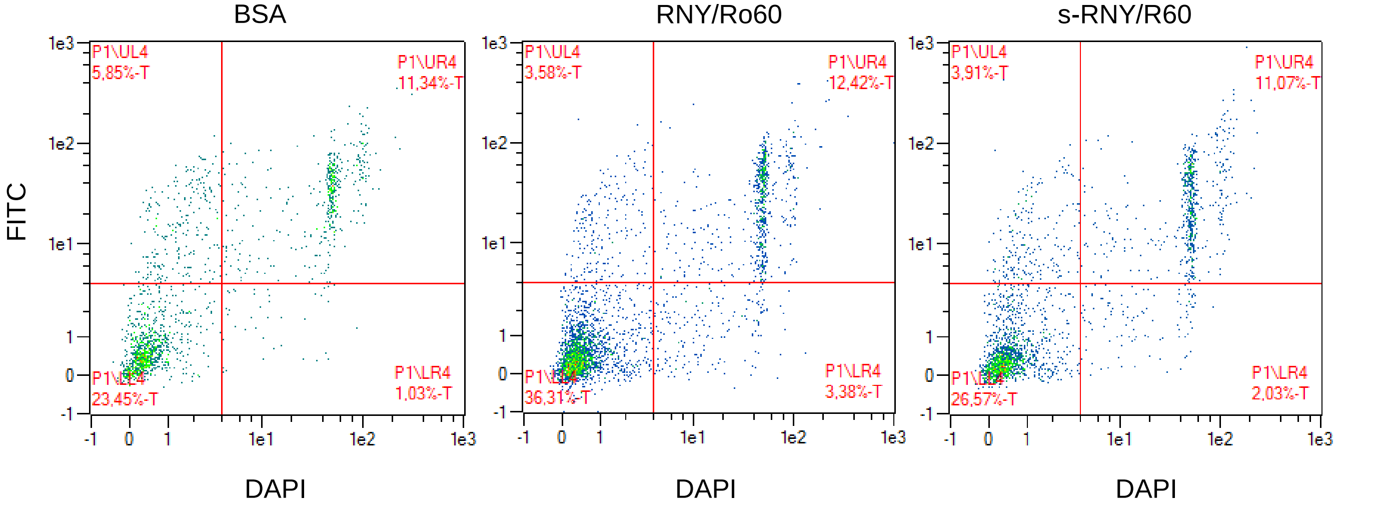

Flow Cytometry

Human Endothelial cells

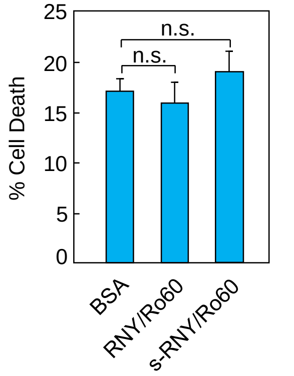

Hizir et al, 2016; Figure S5
